# Community Health Worker Program Sustainability in Africa: Evidence From Costing, Financing, and Geospatial Analyses in Mali

**DOI:** 10.9745/GHSP-D-20-00404

**Published:** 2021-03-15

**Authors:** Patrick Pascal Saint-Firmin, Birama Diakite, Kevin Ward, Mitto Benard, Sara Stratton, Christine Ortiz, Arin Dutta, Seydou Traore

**Affiliations:** a Palladium Group, Washington, DC, USA.; b Palladium Group, Bamako, Mali.; c Independent consultant, Nairobi, Kenya.; d Independent consultant, Washington, DC, USA.

## Abstract

**Background:**

In Mali, community health workers (CHWs) deliver essential community care (ECC) to rural populations. The dominance of external funding for the program threatens the sustainability of this critical workforce as donor financing decreases. This article summarizes results of analyses aimed at assisting Mali's decision makers and leaders in initiating a transition to a sustainable CHW program supported by domestic funding through strategic and rational investment.

**Methods:**

Data on ECC implementation norms, workforce, coverage, utilization, cost, and geospatial features were collected between 2016 and 2019. The data informed interlinked CHW financing analyses—situational, services costing, efficiency, and geospatial mapping. Analysis showed distribution of reported expenditures, estimates of required CHW funding, cost-saving options, and spatially visualized discrepancies between spending estimates and normative costs.

**Results:**

Thirteen financing sources contributed to CHW program expenditures, 88% of which were from international donors, for a package of 23 curative, preventive, and promotive interventions. In 2015, the CHW program spent US$13.01 million; an estimated US$8.36 million would have been needed to achieve the same service volume under standard care protocols. Medicines and start-up training had US$6.88 million more than needed; supervision, program management, and recurrent training components were underfunded by US$2.2 million. Cost-saving opportunities of US$6.16 million were identified in 41 of 44 districts. Funding reallocation opportunities (after meeting technical efficiency requirements) were identified in 20 of 44 districts (US$2.56 million). Use of geospatial targeting and mapping suggests district- and village-level reallocation options for theoretical funding surpluses.

**Conclusion:**

CHW costs can be significantly reduced without sacrificing service technical quality. Spending can be geographically targeted to optimize service use by rural populations. Efficiency analyses provide evidence to build stronger engagement, support improved decision making, efficiently prioritize resources, and target investments for sustainable financing of CHW programs.

## Abstract (precis)

Understanding specific program costs through efficiency analyses and geospatial targeting allows national stakeholders to make strategic, targeted investments, making the first steps toward sustainability. Costs required for community health worker programs can be reduced without sacrificing quality, and spending can be geographically targeted to optimize service use by rural populations. Results from Mali provide an example for other sub-Saharan African countries.

## Teaser

**Key Findings:**

- In 2015, cost-saving opportunities of US$6.16 million were identified in 41 of 44 districts.
- Costs required for community health worker (CHW) programs can be reduced without sacrificing quality and spending can be geographically targeted to optimize service use by rural populations.

**Key Implications:**

- Program managers and stakeholders should use geospatial analyses to reflect critically on CHW resource planning and make evidence easier to act upon.
- Key decision makers should assess what efficiency gains in funding can be achieved with geospatial targeting and mapping.

Résumé en français à la fin de l'article.

## BACKGROUND

The equitable provision of critical services to all segments of the population is an ongoing challenge around the world. Health system leaders are increasingly challenged to look beyond their clinical frameworks and find approaches and models that further expand the services they provide outside of the conventional hospital setting. Community health systems (CHSs) provide an alternative to traditional facility-based health systems through a set of local actors, relationships, and processes supporting health at community and household levels. Community health workers (CHWs) have been the cornerstone of CHSs playing a crucial role in providing preventive, promotive, and curative health services to local communities. Countries worldwide seek to leverage the skills, community knowledge, and cultural competency that CHWs can bring, connecting those most at risk for poor health outcomes with the formal health system.^1^^–^^5^ Furthermore, CHW programs are a proven, cost-effective approach compared to conventional health provider-based service delivery models.^6^^–^^7^

With nearly 60% of its population living in rural areas,^8^ sub-Saharan Africa relies on CHWs as a cost-effective alternative to traditional facility-based service delivery approaches. However, the scarcity of domestic funding for these programs hampers financial sustainability. Sub-Saharan African countries benefited from high (70.2%) development assistance disbursed to CHW-targeted projects between 2007 and 2017, with external donors accounting for nearly 46% of the average annual total funding amount.^9^

For the past 3 decades, Mali's community health system has played a significant role in determining the supply of and demand for community-level health services. Populations across the country ensure that key health services are available in communities through public-private partnerships. Community-level health service delivery is built on a network of privately owned nonprofit community health centers (CHCs) founded by communities and managed by elected community health boards (CBs).^10^ Through a public-private partnership agreement between the central government and CBs, the Ministry of Health and Social Affairs (MOHSA) provides technical supervision and other support and the CHCs provide public health services to their catchment area. The National Federation of Community Health Boards (NFCB) was created as a central body providing regulatory oversight over and management support to CBs. The first CHC was created in 1989^10^; as of December 2018, there were 1,368.^11^ The Malian government introduced CHWs to strengthen rural health care delivery with the support of international partners, following the 2009 census findings that mortality ratios for mothers and children (aged 12–59 months) were respectively 5 and 2 times higher in rural areas.^12^ Since then, CHWs have been delivering ECC to rural populations (i.e., those living more than 5 km from a CHC). The CHW program is funded primarily by international donors, heightening concern for the sustainability of this critical workforce as donors announce decreases in financial support.

**Figure:**
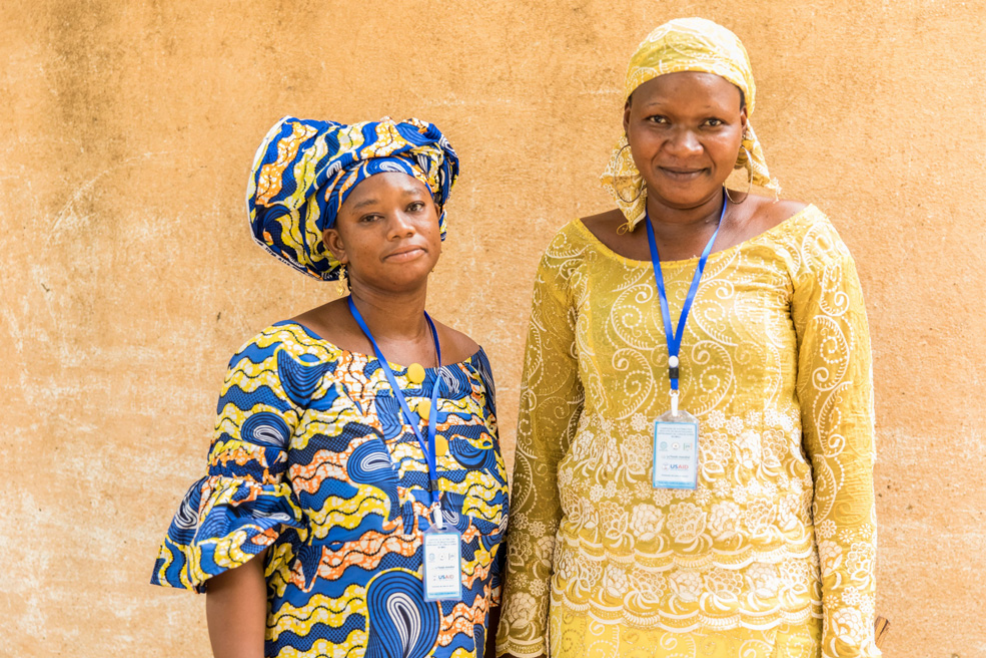
Community health workers in Mali. © 2018 Souleymane Bathieno/Health Policy Plus

Efficiently run CHW programs are vital in resource-limited health systems. Providing evidence to guide rational use of scarce public funds can catalyze country governments' transition to greater domestic funding and contribute to their “journey to self-reliance.”^13^ However, economic analyses focusing on technical and allocative efficiencies of CHW programs in sub-Saharan Africa to inform sustainable domestic investment approaches are rare. Unreliable information about the number, location, terms of service, employment prerequisites, and modes of payment of CHWs in Mali make planning difficult. Lack of cost data further hinders adequate domestic financing of CHW programs and efficient use of available resources as communities and government entities could not rely on accurate data on the real cost of service provision components, including human resources (salary and benefits), drugs and supplies, pre- and in-service training, supervision, and other program management costs. Part of this challenge is explained by the absence of a central repository with information on financing sources for remunerations, equipment, and other program funding areas. This article describes sets of analysis conducted in a step-wise manner that holistically examine national-level CHW financing to help Mali's decision makers initiate a realistic and sustainable program transition to domestic funding by strategic and rational investment.

Providing evidence to guide use of public funds can catalyze country governments' transition to greater domestic funding and contribute to their “journey to self-reliance.”

## METHODS

The U.S. Agency for International Development (USAID)-funded Health Policy Plus (HP+) project conducted the following interlinked analyses—situational, costing, efficiency, and geospatial mapping—between 2016 and 2019. These analyses covered 5 regions in southern Mali that implemented the CHW program, gathering 2015 program data.

### Objectives

The situational analysis defined sustainability concerns raised by USAID/Mali as it prepared to decrease its support to CHWs. The situational analysis had the objective of identifying CHW program components and determining the distribution of reported expenditures and related patterns. The costing analysis followed and had the dual objectives of estimating the necessary CHW funding based on national standard care protocols and examining required program financing changes. Lastly, the combined efficiency and geospatial mapping analyses had the objectives of identifying potential cost-saving options through efficiency improvements, quantifying and spatially visualizing discrepancies between spending estimates and normative costs, and identifying geographic areas for better-targeted funding.

### Type of Data Collected and Sources

Information was collected in-country between 2016 and 2018 using semistructured key informant interviews, questionnaires, consultative meetings with community health experts and secondary information sources. The study team used Microsoft Excel-based templates to obtain expenditure data, service input costs, and utilization data. HP+ designed questionnaires that were administered through semistructured key informant interviews to explore the landscape, opportunities, and challenges of the CHW program.

Key informants were purposively selected based on their institution's role in the CHW program such as funding, implementation, advocacy, norm-setting, management, coordination, and policy responsibilities. Experts were chosen based on their ability to exercise legitimate authority over or influence on one of the following: the choice of CHW location, recruitment and training needs, stakeholder coordination, key program management functions (supply of medicines and equipment, statistical reporting, and supervision), and regulatory and normative decisions. Table 1^14^ summarizes the types of information collected from primary and secondary sources—more detailed information on type of data used and a profile of experts are available in a Supplement.

**TABLE 1.** Summary of Type of Data Used, Sources, and Collection Periods, Interlinked Analysis of Community Health Worker Programs, Mali

Type of Data and Collection Period	Primary Sources	Secondary Sources
**CHW situational analysis (September 2016 to February 2017)**		
**Program level (regions and health districts)** - Population breakdown, health system levels, numbers and geographic distribution of CHWs, CHW financing sources and amounts spent, ratios of CHWs per population and community or household	- Questionnaires and data collection sheets filled by: National Statistic Office, National Health Directorate, MOHSA Division of Equipment and Finance, CHW program implementing partners, Regional Health Directorates, local authorities - Interviews with ECC managers, head of Health Facilities Regulation Division, Drug and Pharmacy Director, health district managers	- Baseline year reports from CHW implementing partners, local health information system, national health accounts, ECC national reviews, regional ECC managers - Mali Demographic Health Survey (fifth edition) - Health area microplanning monitoring guide (2014)
**Costing CHW provided services (February to April 2017)**		
**Country-level (baseline year)** - National and sub-national population figures and growth rate, gender/age breakdown, per household - Annual inflation rate, currency exchange rate **CHW specific inputs** - CHW cadre information & training, supervision & program management - ECC package, number, and types of services delivered - Standard treatment guidelines - Equipment, medicine, and capital costs **Capital costs** - Description of assets, expected quantities, replacement frequency, and costs **Standard treatment guidelines** - Description, target population, time per service, and quantities of tests, medicines, and supplies	- Interview with head of statistics office of Mali National Statistic Office - MOHSA human resources directorate records - Consultative meetings with expert panel	- Mali general population census 2009 - National ECC implementation guide (Dec. '15) - MOH district-level records, local Health Information System report for baseline - Mali Demographic Health Survey 2013, 2018 - Government of Mali reference price listing for goods and services for 2015 - CHW standardized treatment chart for childhood sickness (June 2016)
**District-level cost efficiencies, geospatial mapping, and analysis (March to June 2019)**		
**Calculated estimates** - Normative cost per CHW service - Number of services per person in target population by type of service in baseline year	Authors	N/A
**Geospatial data** - Geocoded villages; administrative boundaries for health districts, municipalities, and communes; population	N/A	- United Nations Office for the Coordination of Humanitarian Affairs Humanitarian Data Exchange Portal - Mali National Health Directorate GIS database
**Geospatial mapping outputs** - Thiessen polygons for CHW covered villages - Point, choropleth, and Euclidean distance maps	Authors	N/A

Abbreviations: CHW, community health worker; ECC, essential community care; MOHSA, Ministry of Health and Social Affairs.

### Data Analysis

#### CHW Situational Analysis

The situational analysis identified and examined CHW program information related to workforce, health services provided, funding sources, and expenditures. Entry, cleaning, and aggregation of data were conducted manually using Microsoft Excel. The quantitative depiction of the data using descriptive statistics, presented through tabulation and graphs, included (but was not limited to) the number of active CHWs, location, remuneration, and reported program expenditures by location, funding source, and category.^15^ Workforce and program expenditure information synthesized from this analysis provided the basis for further exploration through cost modeling and geospatial mapping.

#### Normative Costing of CHW Service Package

Identifying efficiency opportunities and related financial implications requires comparing actual expenditures to the normative cost of delivering the CHW service package in compliance with national standard care protocols. To derive the normative cost of each service, we analyzed national norms and standards, calculated direct costs of the resources or "ingredients" required for each service (a “bottom-up” activity-based approach), and allocated indirect costs in proportion to the share of CHW time spent on each service (a “top-down” approach). This provider perspective approach to calculating cost per service focuses on the supply-side dimension of service quality. Provider communication, bias, and other key aspects of service quality from the client's perspective client were excluded.

The national ECC implementation guide defines the ECC package provided by CHWs. We analyzed its composition by identifying individual services and their link to public health programs. Building from the expenditure information identified in the situational analysis, we traced the main program elements with identifiable costs needed to deliver services. Labor, medicines, and supplies were classified as direct costs entirely attributable to service delivery. Management, supervision, equipment, and training were considered indirect costs incurred regardless of whether service delivery occurs and cannot be assigned solely to a specific service. CHW equipment (thermometer, scale, bicycle, etc.) was considered an indirect cost as specific aspects, like length and frequency of use directly attributable to each service, were not measured.

Determining a standard level of inputs for the ECC package provided by CHWs involved direct measurement or estimation of the time needed to provide each individual service plus required diagnostic tests, medicines, and supplies. We combined contributions from the expert panel with existing national standard care protocols for child health (a MOHSA document) to determine or confirm standard treatment guidelines and estimate standardized normative inputs required for all ECC services. The CHW standardized treatment sheet for sick children was considered a reference to assess and compare quality of care among children and was designed to encourage efficiency through resource optimization and rational use of treatment inputs. Community health experts confirmed data from nationally published statistics. Information was collected from health authorities and implementing partners on costs of personnel supporting the program (CHWs and managers), expected frequency, and cost of training and supervision.

The analysis unit was cost per service considered from the provider perspective. We analyzed cost using the Community Health Planning and Costing Tool, which estimates unit costs of different program elements (e.g., supplies) per service multiplied by the total estimated number of services.^16^ This approach has been used in more than 15 countries to support analyses for community health investment cases, costing of community health packages, and integrated community case management.^16^^–^^17^ Normative unit costs generated considered likely cost variations among regions for delivering services in more remote locations due to inherent characteristics such as terrain and transportation.

Expected frequency, numbers, and costs of supervision visits and meetings per year were adjusted by region. Travel time and related costs were estimated during meetings with CHW program experts. Fuel forecasts were determined based on expected geographic distances (roundtrip) and fuel consumption per 100 km by type of motor vehicle used. An average percentage mark-up on medicines for transport, storage, management, and distribution was derived from all 5 regions and applied to each unit cost. Capital costs linked to accommodation and working space provided to CHWs by communities were based on a sample of 120 villages in 15 health districts across the 5 regions.^15^ Program expenditure information compiled as part of the situational analysis provided the actual cost data.

#### District-Level Cost Efficiency and Geospatial Mapping Analyses

After calculating the normative cost of each service provided by CHWs, we estimated the technical efficiency of service provision within each health district using program data on the average expenditure per CHW in each region and the number of CHWs active in each district (Table 2).

$${AE}_{D}=\frac{{AE}_{R}}{{No.CHWs}_{R}}\times {No.CHWs}_{D}$$

where AE is actual expenditure, D is district, and R is region.

**TABLE 2.** Overview of the CHW Program in Mali, 2015, from Situational Analysis Results

Region	Koulikoro	Kayes	Mopti	Segou	Sikasso	Bamako District^a^
Target population (total living in rural areas)	1,477,040	961,289	1,181,486	1,281,147	1,622,344	N/A
Population covered by CHWs	869,282	286,779	680,261	712,351	648,353	N/A
Population covered by CHWs, of rural population, %	59	30	58	56	40	N/A
No. villages	1761	1369	1896	2003	1629	N/A
No. villages covered by CHWs	621	256	306	412	591	N/A
No. health districts covered	10	8	8	8	10	N/A
No. CHCs	196	217	168	195	237	N/A
No. CHCs affiliated with CHWs	161	143	146	172	230	
No. active CHWs	526	248	305	448	660	150
CHW of total CHWs, %	23	11	13	19	28	6
No.funding sources	7	7	6	6	6	2
Spending, USD	2,356,633	2,284,933	2,540,402	1,977,545	3,127,960	723,825
Spending of total spending, %	18	18	20	15	24	6

Abbreviations: CHC, community health center; CHW, community health worker.

a Bamako is an atypical district that does not meet any criteria stated in the ECC national implementation guide, both in terms of services provided and the determination of the target population. For more information, please consult the CHW landscape analysis report available at: http://www.healthpolicyplus.com/ns/pubs/7153-7273_MaliSituationalAnalysisJuly.pdf

We calculated the normative cost of providing all services to the CHW-covered population in each district. This calculation used district-level service volumes reported by program implementers in 2015 and the normative cost per service for each of the 23 services. The normative cost for the covered population represents the efficient cost of providing all services delivered by CHWs in 2015 while complying with the MOHSA's national quality standards.

$${\text{NC}\mathrm{for}\text{Covered}\mathrm{Population}}_{D}=\sum_{S=1}^{23}{(\text{No}.\text{Services}\mathrm{Provided}}_{D,S}\times {\text{NC}}_{S})$$

where NC is normative cost, D is district, S is service.

The difference between normative cost and actual expenditure is the technical efficiency gap—how much money was spent beyond or below the normative cost.

$${\text{Technical}\mathrm{Efficiency}\text{Gap}}_{D}={AE}_{D}-{\mathrm{NC}\mathrm{in}\text{Coverage}\mathrm{Area}}_{D}$$

where AE is actual expenditure, NC is normative cost.

The normative district-level cost of full rural population coverage included the normative costs per service from the Community Health Planning and Costing Tool application, the size of the rural population, and the number of services provided per person in covered villages. The normative service cost calculation for the full rural population is based on 3 key assumptions: (1) all individuals in villages covered by CHWs who seek care would be able to access a given service; (2) current CHW service volumes meet demand (defined as the individuals in need using the services) within the covered population; and (3) the volume of services used per person would be the same within the noncovered rural population as in the covered population if CHWs were available.

$${NC\text{for}\mathrm{Rural}\text{Population}}_{D}=\frac{{NC\text{for}\mathrm{Covered}\mathrm{Population}}_{D}}{{CoveredPopulation}_{D}}\times {RuralPopulation}_{D}$$

where NC is normative cost, D is district.

The allocative efficiency gap is the difference between actual expenditure and normative cost to cover the total rural population. This value represents each health district's funding surplus or deficit after the technical efficiency gap has been addressed.

$${AllocativeEfficiencyGap}_{D}={AE}_{D}-{NCforRuralPopulation}_{D}$$

where AE is actual expenditure, NC is normative cost.

We analyzed the geographic distribution of community health resources for the baseline year (2015) and its relationship with normative costs using a GIS tool developed between May 2017 and March 2018 by Palladium as a part of the HP+ project.^18^ The GIS tool allowed for geospatial mapping of district-level program expenditures, district-level costs for full rural population coverage, and village-level CHW coverage. Regional program expenditures were allocated to districts in proportion to the number of CHWs in each.

The types of information needed to create maps for geospatial analysis included geocoded villages; administrative boundaries for health districts, municipalities, and communes; population; 2015 district-level program expenditure; and 2015 district-level normative cost of services used by the population. We compared expenditures supporting population use of the ECC package with corresponding normative costs to provide a visual representation of how opportunities for technical and allocative efficiencies are geospatially distributed. Maps and spatial derivatives were generated using Quantum GIS.^19^

## RESULTS

### CHW Situational Analysis

In 2015, the CHW program spent US$13 million to support 2,337 active CHWs affiliated with 84% of CHCs assigned to more than 2,000 villages across 44 health districts in the 5 southern regions of Mali plus the Bamako District. The program provided access to CHW-provided services to more than 3 million people living in rural areas. Thirteen different financing sources contributed to overall expenditures, 88% from implementing partners funded by international donors. Program expenditures and the number of active CHWs varied across regions (Table 2), although amounts spent between regions do not necessarily follow the number of CHWs. Three regions accounted for 70% of the active CHW workforce but just 57% of program spending.

### Costing of CHW-Provided Services

According to the national ECC implementation guide, CHWs offered 23 curative, preventive, and promotive interventions. Roles and responsibilities were defined, and guidelines were provided to ensure that delivery of these services, supervision, and reporting are integrated as a package across 5 public health programs. The ECC package is linked to services under community mobilization/behavior change communication, nutrition, reproductive health/family planning, malaria, and maternal and child health. Direct and indirect labor costs for CHWs contributed to 20% of total cost per service. Activity reporting was the most labor-intensive and expensive “service” with 95% of its cost attributed to indirects. Management of moderate acute malnutrition was the second most expensive service due to high supplies and medicines costs because of extensive use of ready-to-use supplementary food (RUSF) for child nutritional rehabilitation (Table 3). Results for all services are available in a Supplement.

**TABLE 3.** Costing CHW-Provided ECC Services (Selected Outputs), Mali, 2015

	Coverage of needs,^a^ %	Unit Cost Breakdown (US$)			Total time required to provide services,^b^ hours	Time available spent on service,^c^ %	Total cost, US$ (Share of total cost, %)
		Cost per service	Total Direct cost	Total indirect cost			
**Curative services**							
Malaria rapid screening test	31.4	3.82	0.85	2.97	55,899	1.28	639,847 (8)
Uncomplicated malaria management	4.0	6.76	3.49	3.26	44,992	1.03	829,141 (10)
Moderate acute malnutrition management	33.6	33.84	24.94	8.90	36,916	0.85	1,249,206 (15)
**Preventive services**							
Newborn follow-up	5.6	3.07	0.11	2.97	26,954	0.62	248,554 (3)
Education on lactational amenorrhea method	6.8	3.84	0.13	3.71	5,224	0.12	48,173 (1)
Oral contraceptives (pill) provision	6.8	4.68	0.97	3.71	6,459	0.15	72,578 (1)
Provision of contraceptive injections	6.8	1.14	0.40	0.74	4,868	0.11	66,600 (1)
**Promotive services**							
Home visits	66.4	6.15	0.21	5.93	80,188	1.84	739,212 (9)
**Monitoring and evaluation**							
Activity reporting	N/A	74.82	3.62	71.20	209,952	4.8	1,963,577 (23)

**Figure:**
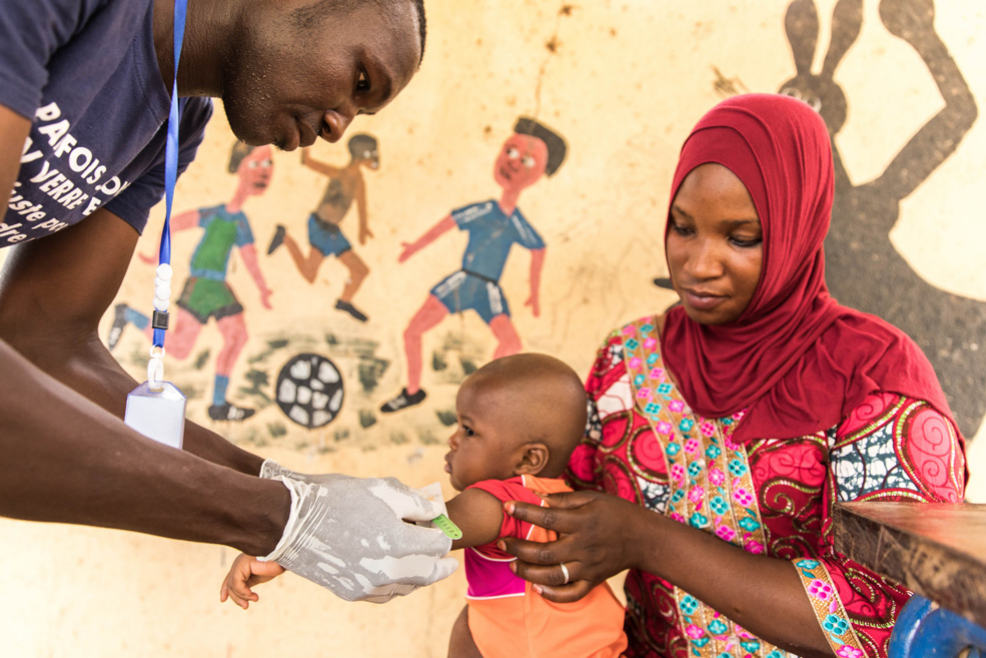
Community health worker in Mali conducts growth monitoring of child. © 2018 Souleymane Bathieno/Health Policy Plus

The CHW program spent US$10.50 on average per service in 2015 to provide 1.24 million ECC services, 55% more than the estimated US$6.80 on average per intervention that would have been needed to achieve the same service volume if standard care protocols were followed which would reduce aggregate spending by 36% (US$8.36 million). The number of ECC services provided per CHW in 2015 varied significantly across districts, with estimates ranging between 94 to 2,287. The proportion of CHW total time available for ECC services in a year spent on direct service provision also varied widely between districts, from 3% to 43%. Comparably low shares of time spent on direct service provision were observed in similar work on integrated community case management programs in other sub-Saharan African countries.^20^ The CHW-to-population ratio ranges from 1 CHW to 702 to 3,478 people per CHW across districts. Funding required for ECC per CHW per year, independent of the quantity of service provided, was estimated at US$2,422 per CHW per year and represented the fixed-cost portion of the program (salaries, supervision, training, and other similar costs). The cost of medicines and supplies varied with the number of services provided, ranging from US$51 to US$3,035 per year per CHW across the 44 districts. Total program normative cost per CHW to provide the services reported in 2015 ranged between US$2,473 and US$5,457 (the amount that theoretically should have been spent if service delivery had followed nationally established normative guidelines). Aggregating these estimates suggests that a CHW would provide, according to norms, on average 566 services at US$3,822 per year, using 17% of one's total available time on ECC services.

**FIGURE 1:**
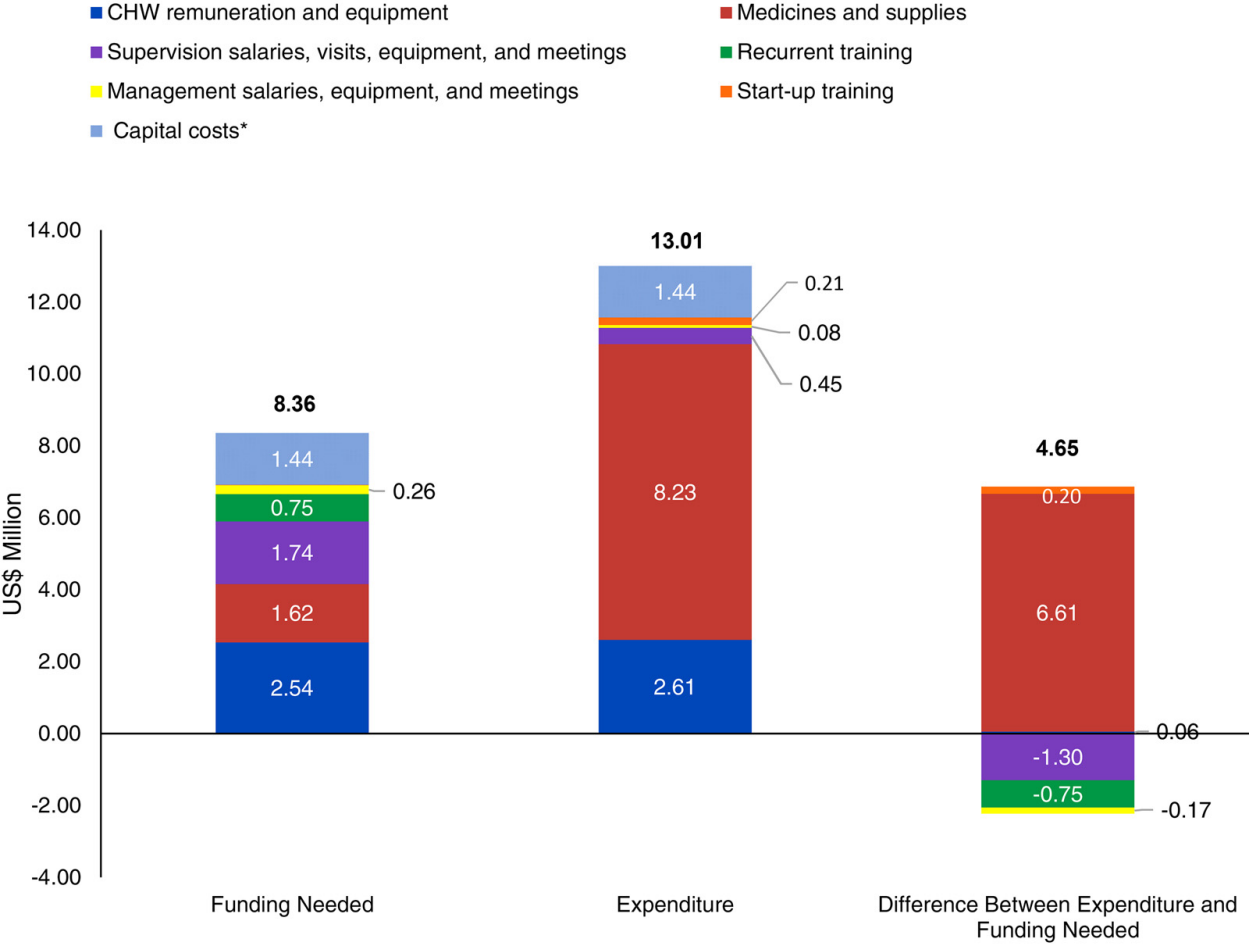
Funding Needed Versus Expenditure by Community Health Worker Program Input in 2015, Mali Abbreviation: CHW, community health worker.*Capital costs include cost of providing living and working space to CHWs at the village level.

Some areas of the CHW program (defined as areas with quantifiable costs related to an identifiable source) benefited from a funding surplus, while others faced a deficit. Figure 1 compares the funding needed in 2015 across program inputs if standard care protocols had been followed, with corresponding spending allocations reported by funding sources. Program input areas such as medicines and supplies, and start-up training had more funding than needed. The excess was estimated at US$6.88 million, 76% of which can be attributed to RUSF and related commodities. Supervision, program management, and recurrent training components were underfunded by US$2.2 million.

### Geospatial Results

**FIGURE 2:**
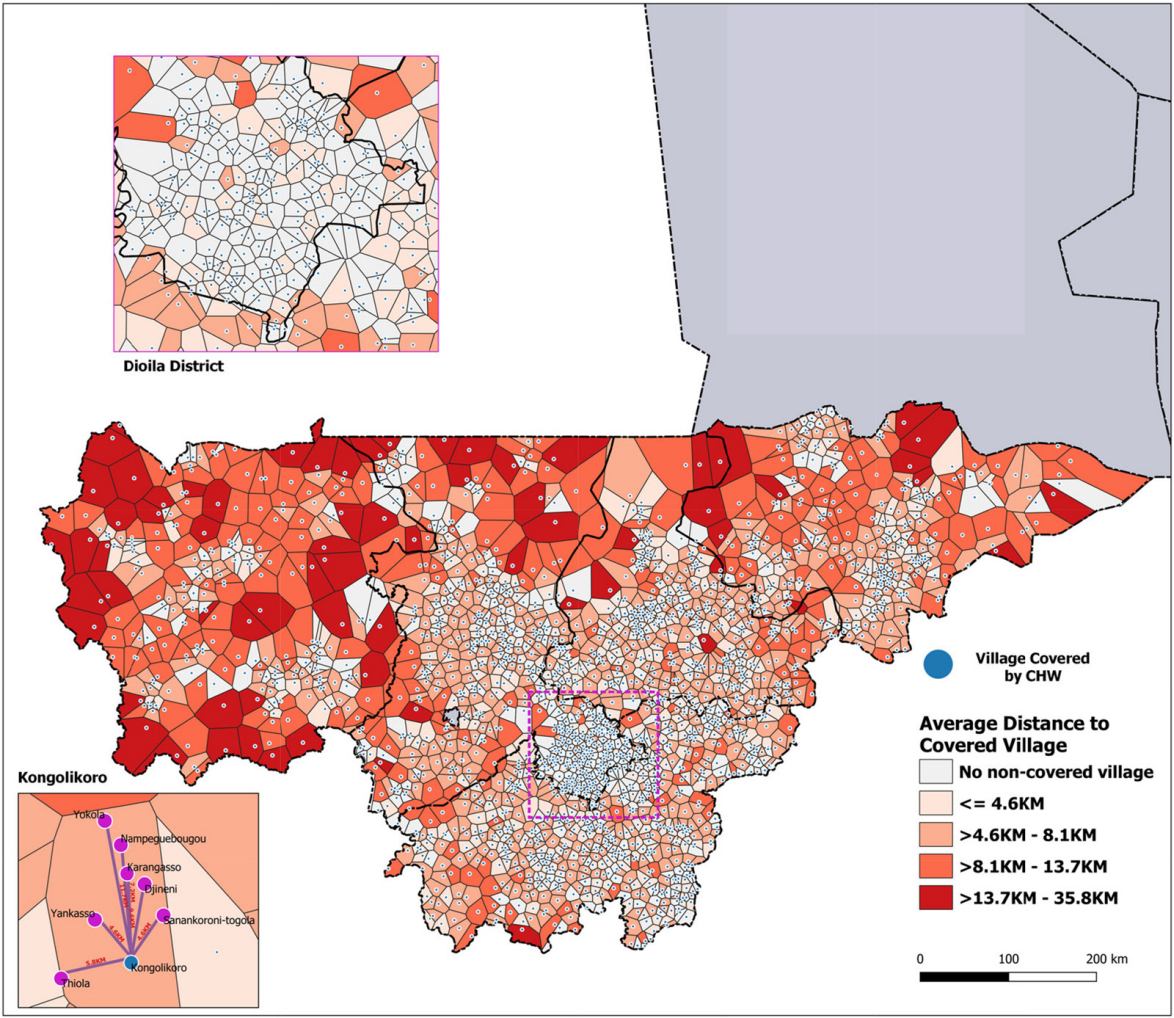
Proximity of CHW-Covered Villages to Non-Covered Villages and Distribution of Non-Covered Villages in 2015, Mali Abbreviation: CHW, community health worker.

Understanding the geographic distribution of CHW program inefficiencies and funding to need misalignments is important to assess what efficiency gains can be achieved with geospatial targeting. In Figure 2, Thiessen polygons are drawn around each covered village. Any noncovered village located within the same polygon shares the same nearest covered village. Shading in the choropleth map represents the average Euclidean distance that noncovered villages are to the closest covered village. Darker shaded polygons represent areas where the nearest covered village is further away, with distances ranging from 13.7 km to 35.8 km. Lack of access in these areas could indicate a greater need for resources to support new CHW locations, by better targeting available CHW resources to increase coverage and move darker shaded areas toward a lighter color indicating improvement.

Significant cost-saving opportunities exist if CHW services implement technical efficiencies by adhering to published norms and guidance from the MOHSA (which we call normative spending).

In most districts, a significant portion of the rural population is not covered by a CHW. Providing services to the currently covered rural population at normative costs could free up funding to extend services to the remaining rural population (Table 4). Technical and allocative efficiency gaps for each health district within the Kayes region, illustrated via red and green cells, present opportunities for reallocation of theoretical funding surplus. Calculations for the 5 regions are available in a Supplement.

**TABLE 4.** Technical and Allocative Efficiency Gaps in Kayes Region, Mali, 2015

Health District	Rural Population [A]	Covered Population [B]	Number of CHW [C]	Average Spending per CHW, US$ [D]	Actual Spending on Covered Population, US$ [E] = [C] x [D]	Normative Cost for Covered Population, US$ [F]	Technical Efficiency Surplus (Deficit), US$ [G] = [E] − [F]	Normative Cost for Rural Population, US$ [H] = [F] / [B] x [A]	Allocative Efficiency Surplus (Deficit), US$ [I] = [E] − [H]
Kayes	210,228	61,361	45	9,213.44	414,604.77	196,305.62	218,299.15	672,559.72	(257,954.96)
Bafoulabe	77,332	33,009	34	9,213.44	313,256.94	99,135.01	214,121.93	232,249.03	81,007.91
Diema	99,199	36,222	27	9,213.44	248,762.86	184,383.31	64,379.55	504,959.41	(256,196.55)
Kenieba	106,732	18,313	14	9,213.44	128,988.15	46,319.44	82,668.71	269,959.42	(140,971.27)
Kita	267,635	82,779	82	9,213.44	755,502.02	117,566.54	637,935.49	380,107.51	375,394.51
Nioro	102,444	14,554	8	9,213.44	73,707.51	36,937.50	36,770.02	259,998.97	(186,291.46)
Yelimane	42,043	14,584	13	9,213.44	119,774.71	21,981.85	97,792.86	63,369.65	56,405.06
Oussoubidiagna	55,676	25,957	25	9,213.44	230,335.98	49,890.50	180,445.48	107,011.73	123,324.26

Abbreviations: CHW, community health worker; ECC, essential community care.

a Estimated by dividing the actual number of services provided by the expected number of services.

b Total time required to provide each service is calculated by multiplying the expected time spent on service (in minutes) by the reported service volume for the year. The estimates are then converted in CHW hours.

c Percentage of CHW time available spent on services is calculated by dividing the total time required to provide the services reported for the year and the total time available for providing EC.

**FIGURE 3:**
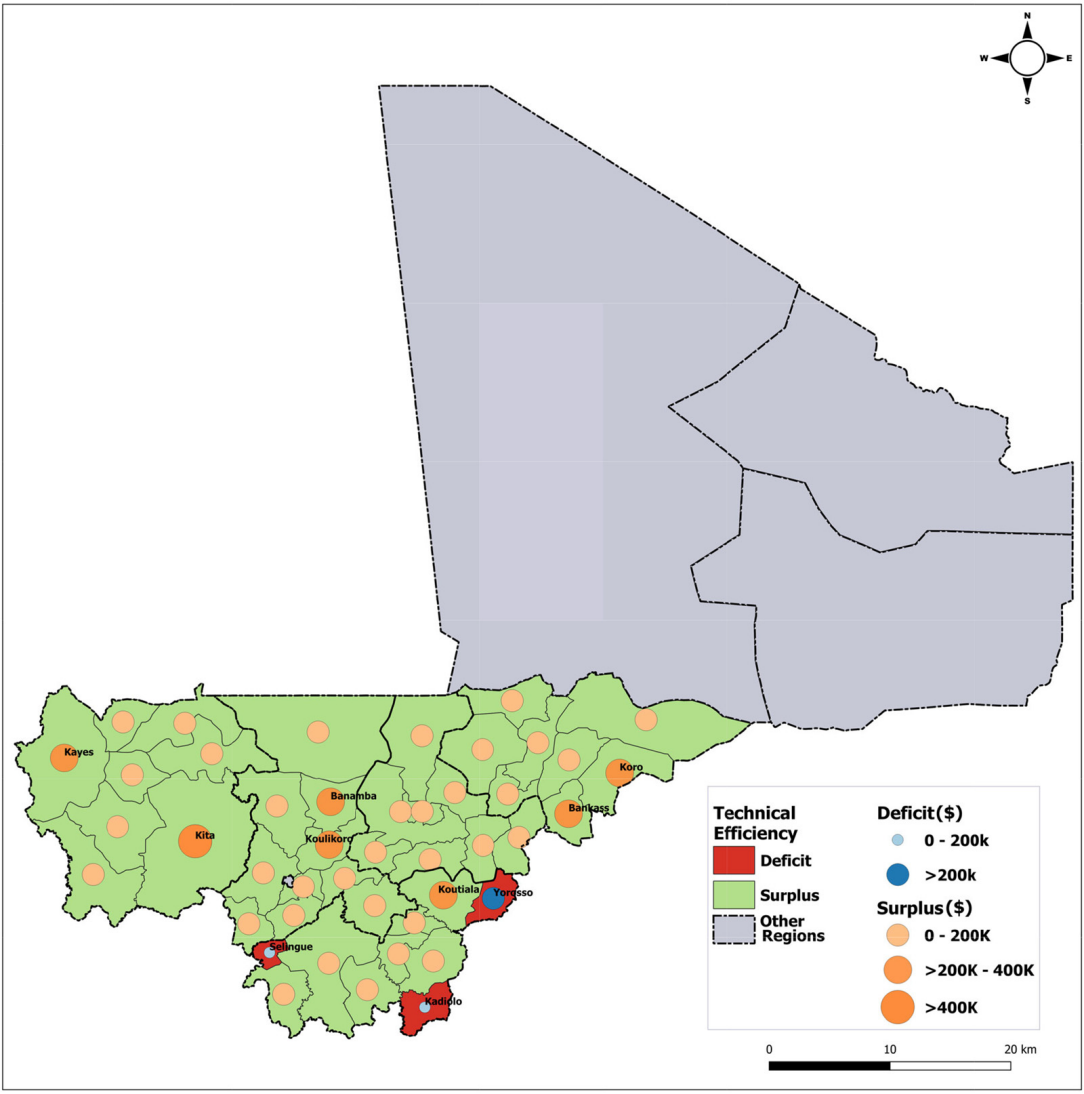
Geospatial Distribution of Technical Efficiency Opportunities for the Community Health Worker Program in 2015, Mali

Figure 3 compares actual district-level spending on services (in US$) delivered with the estimated normative spending to show the magnitude of potential efficiency gains and resource optimization at scale (same population covered and service volume produced). Opportunities for cost saving were identified in 41 of 44 districts (indicated in green in Figure 3), varying in value between US$29,218 and US$637,935 and cumulatively representing US$6.16 million. Repurposing the funding from technical efficiency improvements within districts could cover over 2.1 million more people in rural areas without any redistribution across districts, representing an additional 32.3 percentage points of the rural population and increasing coverage in the 5 regions from 49.0% to 81.3%. Amounts spent by 3 other districts (indicated in red in Figure 3) were lower than their normative spending estimates. Districts in 4 regions spent above what was needed to reach technical efficiency requirements (based on national guidelines) meeting the use of ECC by their covered and total rural population.

Repurposing funding from technical efficiency improvements within districts could cover over 2.1 million more people in rural areas without any redistribution across districts.

**FIGURE 4:**
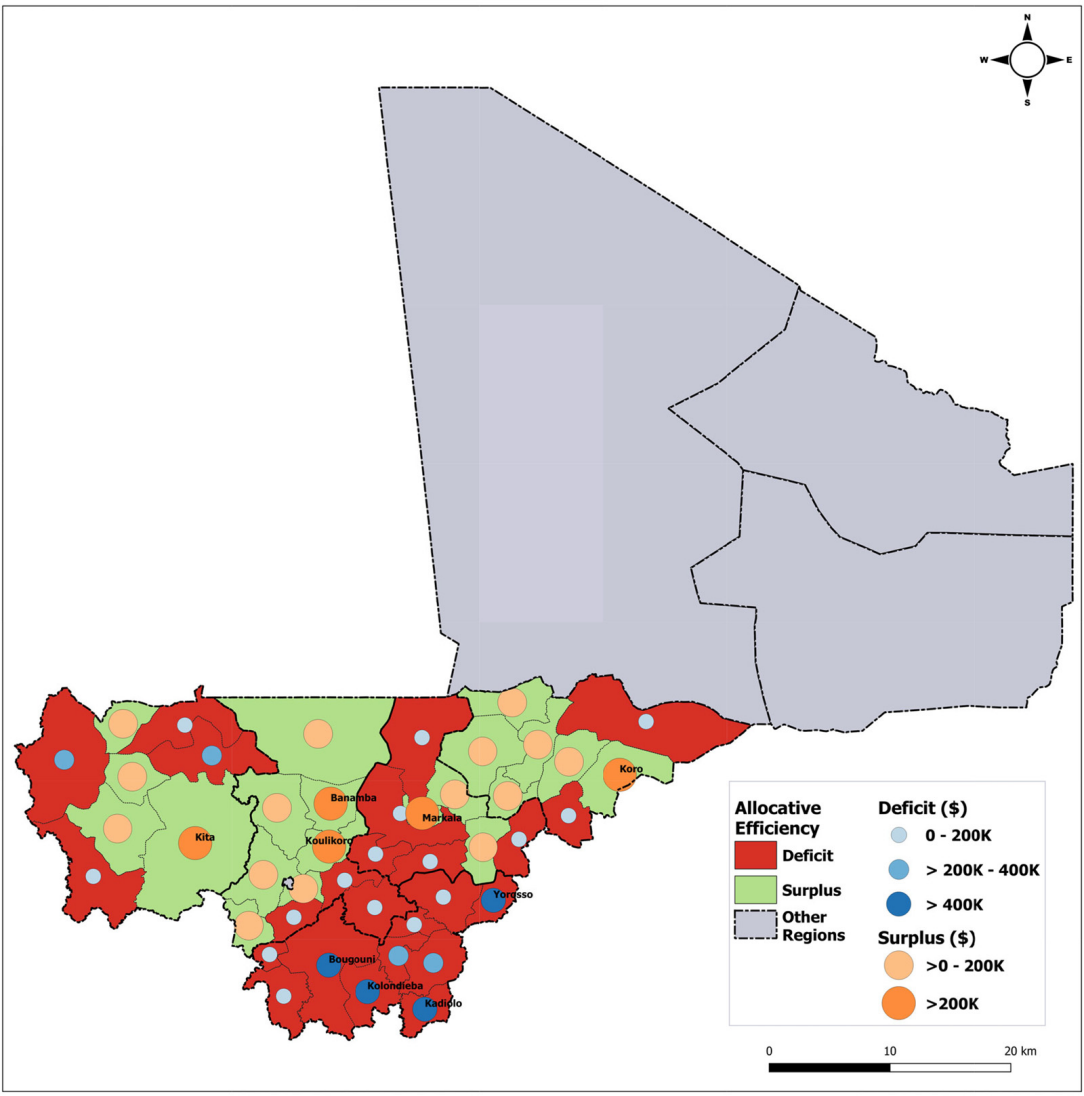
Geographic Distribution of Allocative Efficiency Opportunities for the Community Health Worker Program Across Health Districts in 2015, Mali

**FIGURE 5:**
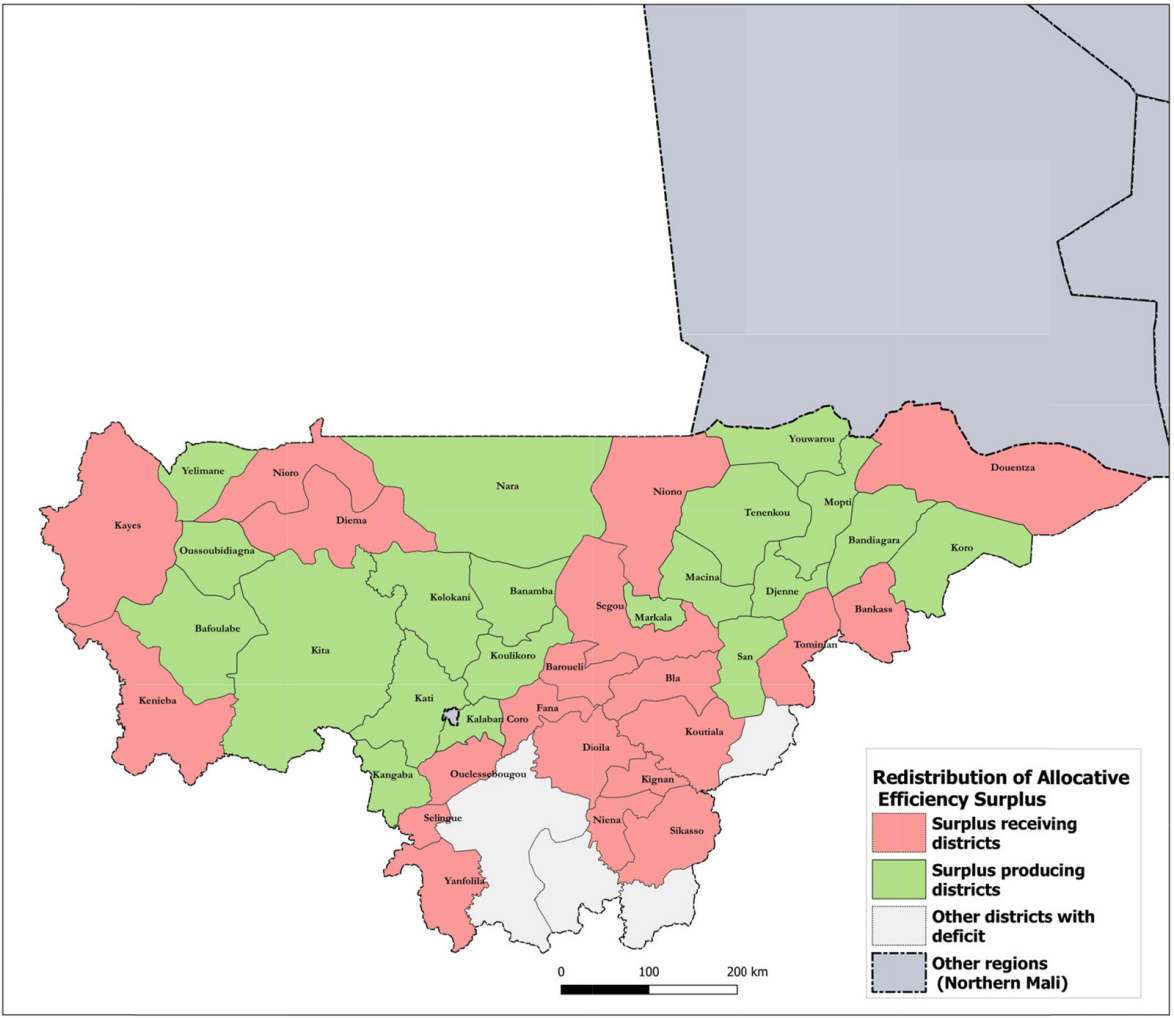
Geographic Redistribution of Community Health Worker Program Theoretical Funding Surplus, 2015, Mali

**FIGURE 6:**
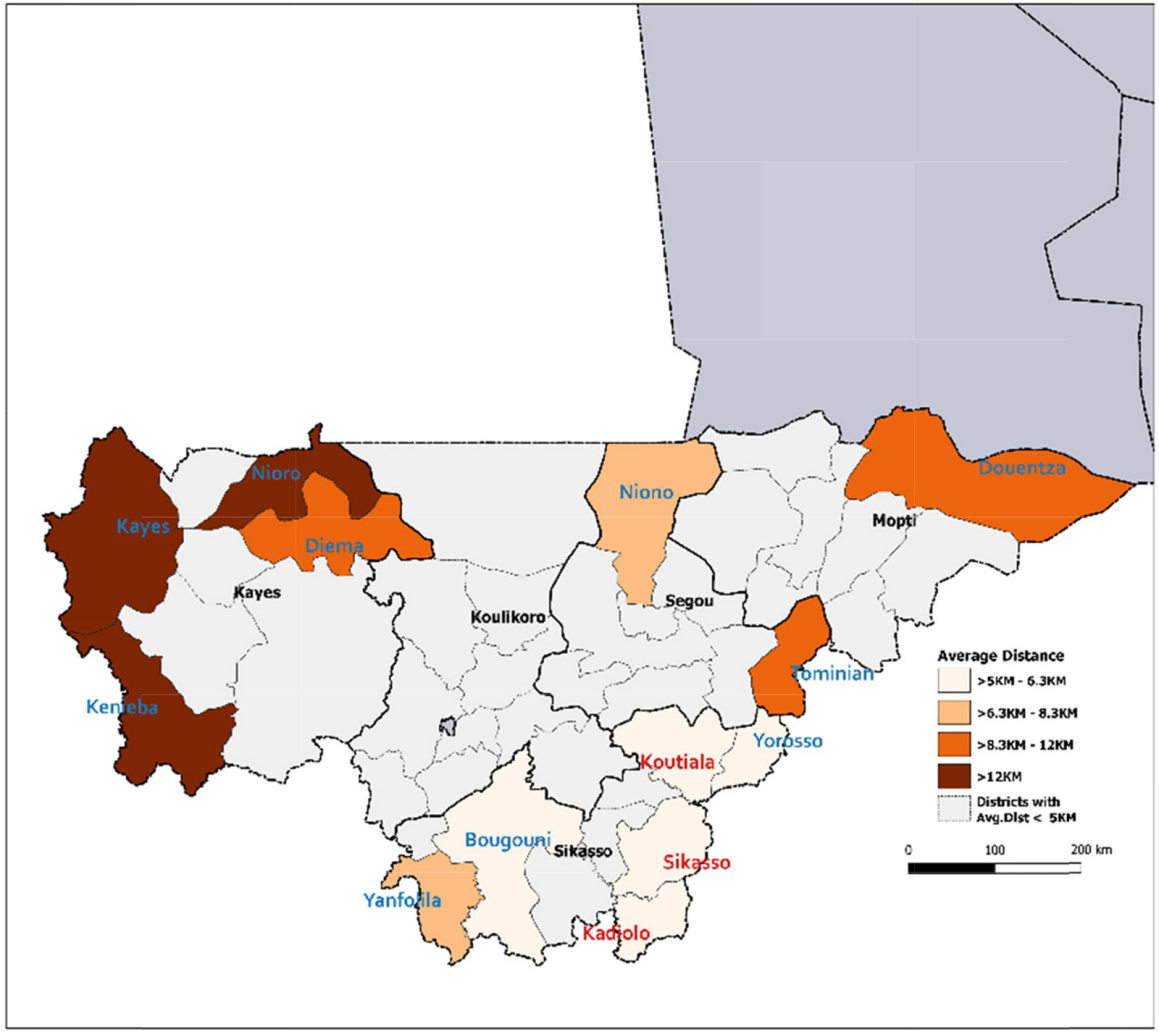
Distance-Based Geospatial Redistribution of Community Health Worker Program Funding Surplus, 2015, Mali

Figure 4 compares actual district-level spending on services delivered to rural populations in 2015 with the estimated normative cost of population use of ECC services to cover the entire district's rural population. Geographic distribution and magnitude of efficiency gaps in US$ are presented across all program districts at scale (same population covered, and service volume produced). Opportunities for funding reallocation were identified in 20 of 44 districts with a surplus between US$8,396 and US$375,395, cumulatively representing US$2.56 million. The remaining 24 districts required additional spending (according to norms) to meet the cost of ECC service use by their rural populations. Deficits across these districts varied between US$511 and US$783,839, reaching a total of US$4.56 million. Figure 5 presents an example of how the theoretical funding surplus available for reallocation can be applied to reach an optimal number of districts supporting the use of ECC services by their entire population (i.e., prioritizing districts with the smallest deficits). In this example, reallocating the funding surplus would bring 20 additional districts to full coverage of their rural population and reach more than 850,000 people, increasing rural population coverage by 13.1 percentage points (from 81.3% to 94.4%). In 13 districts with a funding deficit, 1,637 noncovered villages were located more than 5 km from any point of service (health facility or CHW). We refer to such communities as “isolated villages.” Figure 6 displays the results of prioritizing districts with villages most isolated from the health system, i.e. districts with the farthest average distance between isolated villages and the nearest covered village (based on Euclidean distance). We estimate that this approach would bring 10 additional districts to full coverage, reach rural populations located in approximately 1,468 isolated villages, and increase rural population coverage across 5 regions to 91.6% (Figure 6). The opposite approach—prioritizing districts where isolated villages are located closer to covered villages and are thus easier to reach—would bring 10 additional districts to full coverage, reach approximately 1,278 isolated villages, and increase rural population coverage to 92.1%. Additional maps on point of service distribution and application of geospatial targeting using Euclidean distance mapping analytics are available in a Supplement.

## DISCUSSION

The initial situational analysis shows that compartmentalization of international donor interventions makes the CHW program in Mali vulnerable, exposing leadership and coordination issues as real challenges. Program funding and management information was asymmetric between implementing partners but most importantly between implementing partners and national/local community health stakeholders. None of the 10 (of 13 total) external funding sources identified in the situational analysis supported all program resource areas needed to operationalize CHW activities across all southern regions in 2015.

According to CHW program managers at the MOHSA, choice of CHW program geographic (region or district) or program (for remuneration, supervision) by a donor is discretionary to a donor regardless of what is being provided by others. The mapping process of funding sources for some program areas is another good example of how donor compartmentalization issues are reflected not only in the data collected but also in the approach used to access the information. Nine different funding sources were recorded in 2015 for CHW remuneration and equipment, and 5 for medicines and supplies. This information was not available at central level at the time of the study. Program data including CHW funding source, workforce, and service utilization were gathered directly at the regional level and from implementing partners and donors. Key informants consulted further underscored the lack of information sharing between donors, for example, that an implementer could continue to remunerate CHWs while being unaware that they did not have the necessary medicine and supplies to continue working effectively.

*The big challenge at the moment is the lack of control over the cost and availability of medicines.* —Implementing partner, October 2016

Such challenges result in a wide variety of funders, each having different mandates and interests that are not necessarily aligned with national priorities, leading to investments with no clear transition to the government or another partner, which can also cause temporary suspension of CHW services. MOHSA leadership needs to make decisions to bring expenditures on key program areas closer to less costly normative levels of funding. Reaching this stage of efficiency in programming at the central management level requires coordination and consensus among all funding sources for reprioritizing historic spending allocations according to country-endorsed national standards.

Reflecting on why expenditures on certain program areas were significantly higher than for the normative costs is critical so that implementers or practitioners outside Mali (but in similar contexts) can also use these results. One way of ensuring that limited budgets go further is increasing efficiency, potentially by adhering to national service delivery guidelines. Our results in Mali suggest that service delivery, supervision, and medicine and supply distribution did not fully comply with standards, which led to a waste of resources. Lack of compliance to service standards by CHWs not regularly supervised might have led to input overuse which inflates consumption, making the forecast of expensive medicines and supplies, particularly RUSF, unreliable.

Management of moderate acute malnutrition is the most commodity-intensive service provided by CHWs as evidenced by a cost structure dominated by variable costs with RUSF comprising 73% of the total cost per service. Spending on RUSF is driven by its overall use which is expected to increase proportionally as the volume of services increases. The relationship between spending on RUSF and outputs (cases of moderate acute malnutrition managed) might not be necessarily linear in practice. In addition to potential wastage at CHW level, other factors might affect use of RUSF and other medicines or supplies distributed directly to CHCs by implementing partners. Evidence from Ethiopia has shown that in certain settings micronutrient enhanced commodities can be subject to particularly high levels of leakage and misuse with products ending up for sale in shops.^21^ Limitations faced by CBs and CHCs in Mali are documented in government policy documents and could further explain the cost differences observed. The recently issued Mali Action Plan 2020-2023 states that 24% of CBs are considered not functional and few are capable of conducting effective financial control or transparent reporting of availability and use of resources at CHC level. According to members of the NFCB, implementing partners provide insufficient support to strengthen NFCB's role and authority, preferring to engage directly with CHWs and, to a lesser extent, CBs.

*Some implementing partners sought quick results at the expense of systemic integration and efficiency*. —NFCB member, September 2016

The vertical nature of such approaches has also been echoed by other stakeholders concerned about its negative effects on the relationship between communities and CHWs ultimately affecting ownership of the program.

*CHWs are identified according to their donors …, they are not identified as an integral part of the community.*—Regional Health Office Director, October 2016

Furthermore, 1 high-ranking MOHSA civil servant interviewed indicated that new CHW-covered villages or staff replacements (which drive start-up training costs) were not necessarily controlled or recorded accurately by the government nor reported by implementing partners. This gap in coordination links back to donor compartmentalization issues and leads to asymmetric information between central management of the CHW program overseen by the MOHSA and field operations supported by implementing partners.

National ECC implementation guide and standard care protocols such as the CHW standardized treatment sheet for sick children (Table 1) offer opportunities to increase technical efficiency. The protocols prescribe less costly combinations of inputs while achieving the same number of outputs (or achieving more outputs for the same level of inputs) without sacrificing quality. Creating awareness around the existence and implementation of these norms is key to streamlining efficiency and quality across the CHW program.

Complying with standards can reduce program spending to achieve the same service volume, generating savings to be invested elsewhere in the ECC program. For example, CHW supervision, often considered one of the weakest links in CHW programs (although noted as critical for CHW effectiveness and efficiency),^7^^,^^22^ was severely underfunded in 2015, meeting only 26% of the normative cost required. While donor funding provided adequate total funding for most aspects of the CHW program, we found internal inefficiencies and misalignments of funding. Some areas got more support, as did some geographies. Other areas had access challenges. Norms were not followed, leading to inefficient spending of available resources. Mali could make better use of CHW program funding if guided by data that inform efficient use of domestic resources mobilized, while also adequately resourcing key program areas.

Mali could make better use of CHW program funding if guided by data that inform efficient use of domestic resources mobilized.

Given budget and health workforce constraints in lower- and middle-income countries, CHWs face challenges without receiving the needed support in providing expected services. The productivity differences were discussed with local stakeholders. These discussions generated possible explanations, including the presence of user fees and variability in population density (captured by the population-to-CHW ratio) linked to the mining industry. Although user fees are authorized in Mali, they are known to inhibit access to care in other settings.^23^^,^^24^ Mali is a major producer of mined gold in Africa.^25^ The presence of gold extraction sites may distort population dynamics and affect density as human activity increases significantly in these regions or districts.^26^ Enforcing national protocols for ECC services through an adequately funded supportive supervision system would allow significant cost savings by improving CHWs' ability to comply with technical efficiency requirements that would, in turn, lead to less costly service provision.

Optimizing limited resources through cost savings provides opportunities to increase coverage to currently noncovered populations. Mali geospatial analyses illustrate options for decision makers. Planning can be more engaging by visually supporting identification and prioritization of districts with the highest opportunities for technical efficiency improvements. Using recognizable maps makes advocating for funding surplus reallocations and investments easier. Location data (distance and proximity) used in geospatial targeting help stakeholders reflect critically on CHW resource planning and make evidence easier to act upon. Geospatial analytics and supported interfaces facilitate analytical reasoning for decision makers by turning data into information, information into insight, and insight into practical decision making. Geospatial analytics can further help organizations anticipate and prepare for upcoming changes due to evolving spatial conditions or location-based events such as a community-level response to the COVID-19 pandemic.

Our findings in Mali are relevant to the bigger discussion around system integration and sustainability of CHW programs, given that primary health care is the key to reaching universal health coverage. In Mali, CHWs are essential to delivering that care.^27^ Those cost-saving approaches would allow Mali, with adequate political prioritization by the government, to financially sustain the CHW program. Evidence from HP+ has indicated that the total government budget from domestically generated resources increased between 2015 and 2017 by a 6 percent gross domestic product equivalent (valued at nominal value). In addition to yearly budget increases, most sectors benefited from funding increases through mid-year budget adjustments. Instead, reductions were applied to health and represented the highest budget cut proportions across all government sectors (lower adjusted amounts compared to the initial authorized allocations). Public spending opportunity loss for the health sector was estimated at US$51.8 million (2015 US$) between 2015 and 2017.^28^ This amount could have sustained the entire CHW program at scale for more than 6 years.

**Figure:**
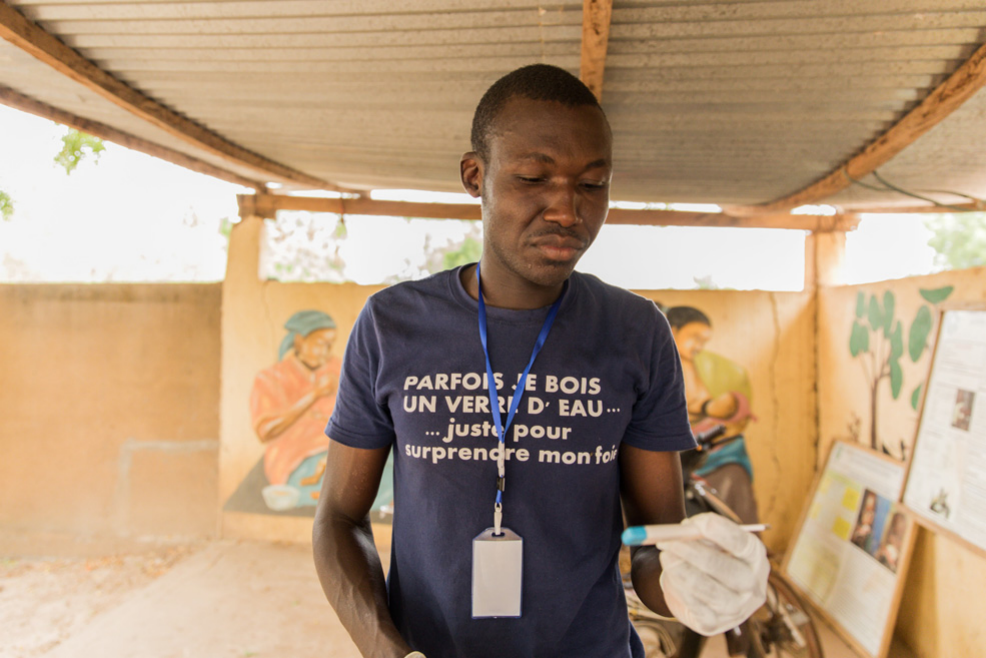
Community health worker in Mali assesses temperature. © 2018 Souleymane Bathieno/Health Policy Plus

Implementing cost-saving approaches would allow Mali, with adequate government prioritization, to financially sustain the CHW program.

International donor assistance is volatile and has inherent limitations, such as being temporary with "short-cycle" characteristics and strings attached.^29^^–^^30^ High dependence on external sources of funding to support CHWs across sub-Saharan Africa and confirmed donor funding cuts in 2019 that targeted Mali CHWs, according to an official unpublished USAID letter, pose a significant threat to the financial sustainability of this frontline workforce. The current funding landscape for CHWs shows a critical need for increased domestic resource mobilization. This landscape warrants increased emphasis on outcome-based results (reflected by improved health indicators) linked to financial and system requirements to sustain CHW programs at scale and better integrate these programs into national health systems. There has been an emerging focus within the international donor community on developing a conceptual understanding of how CHW programs are designed and how they should interface with both formal and community health systems.^31^

Fragmented funding, due in part to limited donor coordination, combined with insufficient government leadership, inhibits sustainable financing and contributes to inefficient spending in many countries.^9^^,^^32^^–^^35^ It hinders CHWs' integration into national health systems, preventing stakeholders from collectively embracing horizontality through harmonized approaches to financial planning, programming, and prioritization. Robust data and results from these types of analyses offer entry points to engage government and communities to invest more in CHWs together. At the central level, findings can demonstrate that costs required for CHW-delivered interventions meeting current rural demand and unmet need for ECC services can be significantly reduced without sacrificing quality. At a local level, results give stakeholders analytical insight and understanding that spending can be geographically targeted to optimize service use by rural populations.

### Limitations

Expenditure information self-reported by implementing partners could not be independently verified. Potential supply-side limitations of the ECC package were not considered. Assessment or recording of time required for delivering each service was made through limited direct observation and estimates relied mainly on expert opinion. Demand-side factors such as financial barriers affecting use of services in CHW-covered areas were not assessed, as information on user fees possibly charged by CHWs for some of the services provided were not collected. Costs did not include health system strengthening activities required to increase compliance with norms and standards of care. Differences affecting use of ECC services were not accounted for between CHW-covered and noncovered villages within the same health district. Elements that might explain the variation in CHW productivity such as workload, adequate supplies and equipment, and acceptance and respect from the community and health systems were not explored in our analyses.^36^ Although a useful proxy in regions where common routes are not always mapped, Euclidean distance does not consider constraints in travel such as road conditions, rivers, and terrain.

## CONCLUSION

Mali's CHW program is hampered by fragmentation of funding and interventions compounded by leadership challenges, noncompliance with national standards of care, and inadequate re-sourcing of key program areas. Results from Mali's case show how efficiency analyses can provide an evidence base to build stronger stakeholder engagement and support improved decision making for CHW financing. Our analyses bring a level of understanding of CHW program costs and challenges that allows the Malian government and other stakeholders to prioritize resources efficiently, and thus afford targeted investments to begin sustainably financing the CHW program.

Evidence presented indicates system and program implementation changes that could be tested to suggest future adaptations. Such changes could further guide operations research in other sub-Saharan African countries to improve community health and the sustainability of CHW programs. Building CHSs from the ground up, while carefully considering local contexts, is essential to inform decisions about where, when, and how care is provided within a community. Our findings can also further contribute to global thinking and local actions around system integration. They can debunk frequent misconceptions which present CHW programs as a unidimensional human resource solution to health care access at the community level without recognizing the dynamics of CHS local actors, implementing partners, and the broader health system.

## Supplementary Material

20-00404-Saint-Firmin-Supplement.pdf

## Translation

**En Français**

**Pérennisation du Programme des Agents de Santé Communautaires en Afrique: Évidences Provenant d'Analyses de Coûts, de Financement et de Données Géospatiales au Mali**

**Principales constatations**

- En 2015, des possibilités de réduction de coût s'élevant à US$6,16 million ont été identifiés au niveau de 41 des 44 districts sanitaires évalués.
- Les coûts nécessaires au programme des agents de santé communautaires peuvent être réduits sans sacrifier la qualité et les dépenses peuvent être ciblées géographiquement afin d'optimiser l'utilisation des services par les populations rurales.

**Implications clés**

- Les gestionnaires de programme et les intervenants devraient utiliser les analyses géospatiales pour réfléchir de façon critique à la planification des ressources du programme ACS et se conformer plus facilement aux actions à entreprendre.
- Les principaux décisionnaires devraient évaluer les gains d'efficience en matière de financement qui peuvent être réalisés grâce au ciblage et à la cartographie géospatiaux.

**RÉSUMÉ**

**Contexte:** Au Mali, les agents de santé communautaires (ASC) fournissent des soins essentiels dans la communauté (SEC) aux populations rurales. La prédominance du financement externe supportant ce programme menace la viabilité de cette main-d'œuvre essentielle à mesure que le financement des bailleurs diminue. Cet article résume les résultats d'analyses visant à aider les décisionnaires et dirigeants du Mali à entamer une transition réaliste vers un programme ASC durable soutenu par un financement national par des investissements stratégiques et rationnels.

**Méthodes:** Des données sur les normes de mise en œuvre des SEC, la main-d'œuvre ASC, la couverture, le coût et l'utilisation des services ainsi que des caractéristiques géospatiales ont été recueillies entre 2016 et 2019. Les données ont éclairé une suite de travaux analytiques interdépendants liés au financement du programme ACS — analyse: situationnelle, du coût des services, d'efficience et géospatiale. Les analyses ont montré la répartition des dépenses déclarées, les estimations du financement requis pour les ASC, les options de réduction de coûts et les écarts visualisés spatialement entre les estimations de dépenses et les coûts normatifs.

**Résultats:** Treize sources de financement ont contribué aux dépenses du programmes ASC, dont 88% proviennent de bailleurs de fond internationaux, pour un ensemble de 23 interventions curatives, préventives et promotionnelles. En 2015, le programme ASC a dépensé US$13,01 millions; US$8,36 millions auraient été nécessaires pour atteindre le même volume de services en vertu des protocoles de soins standard établis par le Mali. Les médicaments et la formation initiale ont bénéficié de US$6,88 millions de plus que nécessaire; la supervision, la gestion de programme et la composante recyclage de la formation ont été sous-financées de US$2,2 millions. Des possibilités de réduction de coût de US$6,16 millions de dollars ont été identifiées dans 41 des 44 districts évalués. Des possibilités de réaffectation du financement (après avoir atteint les exigences d'efficience technique) ont été identifiées dans 20 des 44 districts évalués (US$2,56 millions). L'utilisation du ciblage et de la cartographie géospatiaux suggère des options de réaffectation des excédents théoriques de financement aux niveaux des districts et des villages.

**Conclusion:** Les coûts du programme ASC peuvent être considérablement réduits sans sacrifier la qualité technique des services associés. Les dépenses peuvent être ciblées géographiquement afin d'optimiser l'utilisation des services par les populations rurales. Les analyses de l'efficience fournissent des données probantes pour renforcer l'engagement, appuyer l'amélioration de la prise de décisions, prioriser de façon efficiente les ressources et cibler les investissements adéquats pour un financement durable des programmes ASC.

**Peer Reviewed**
