# Supplementary material for: Community Health Worker Program Sustainability in Africa: Evidence From Costing, Financing, and Geospatial Analyses in Mali
Source: Glob Health Sci Pract. 2021 Mar 15;9(Suppl 1):S79–97. doi: 10.9745/GHSP-D-20-00404 (PMC7971366; doi:10.9745/GHSP-D-20-00404)
Supplement: 20-00404-Saint-Firmin-Supplement.pdf [file 20-00404-Saint-Firmin-Supplement.pdf]

Supplement to: Pascal Saint-Firmin P, Diakite B, Ward K, et al. Community health worker program sustainability in Africa: evidence from costing, financing, and geospatial analyses in Mali. *Glob Health Sci Pract*. 2021;9(Suppl 1). https://doi.org/10.9745/GHSP-D-20-00404

Supplement

Table S1. Type of data used, sources, and collection periods

Type of Data and Collection Period	Primary Sources	Secondary Sources
**CHW situational analysis (September 2016 to February 2017)**		
**Program level (regions and health districts)**<br>- Population breakdown between urban and rural areas<br>- Health system levels—numbers of districts, referral health centers, community health centers, CHW village health posts<br>- Current numbers and geographic distribution of CHWs<br>- CHW financing sources and amounts spent for baseline year by program areas—supervision, management training, equipment, medicines and supplies, capital costs<br>- Actual ratios of CHWs per population and per community or household	- Questionnaires and data collection sheets filled by: National Statistic Office, National Health Directorate, MOHSA Division of Equipment and Finance, CHW program implementing partners, Regional Health Directorates, local authorities<br>- Interviews with ECC national manager, ECC regional and district managers, head of Health Facilities Regulation Division, Drug and Pharmacy Director, health districts managers	- Baseline year reports from CHW implementing partners, local health information system, national health accounts, ECC national reviews, regional ECC managers<br>- Mali Demographic Health Survey (fifth edition)<br>- Health area microplanning monitoring guide (2014)
**Costing of CHW provided services (February to April 2017)**		
**Country-level (baseline year)**<br>- National and sub-national population figures and growth rate<br>- Population breakdown by gender and age groups<br>- Annual inflation rate<br>- Average number of persons per household<br>- Currency exchange rate	- Interview with head of statistics office of Mali National Statistic Office	-Mali general population census 2009
**CHW cadre**<br>- Normative ratios of CHWs per population and household<br>- CHW expected attrition rates<br>- Expected CHW working hours<br>- Salary/stipend and benefits	- MOHSA human resources directorate records<br>- Consultative meetings with expert panel	- National ECC implementation guide (December 2015)<br>- MOH district-level records<br>- Local Health Information System report for baseline year<br>- Mali Demographic Health Survey 2013, 2018<br>- GOM reference price listing for goods and services for 2015<br>- CHW standardized treatment chart for childhood sickness (June 2016)
**Supervision and program management**<br>- Normative ratios of CHWs per supervisor and manager<br>- Expected time for CHW supervision and program management<br>- Salary and benefits<br>- Expected frequency, numbers, and costs of supervision visits and meetings per year<br>- Expected frequency, numbers, and costs of management meetings per year		

Type of Data and Collection Period	Primary Sources	Secondary Sources
**ECC package, number, and types of services delivered**<br>- List of services provided by CHWs grouped by health area/program type<br>- Type of services – counseling, malaria treatment, referral, and other.<br>- Target population group for each service<br>- Incidence, prevalence, and expected utilization rates for each service<br>- Actual number of services provided in the baseline year of analysis		
**Training**<br>- Training session types (for CHWs, supervisors, and managers), expected frequency, number of participants, and costs		
**Equipment**<br>- Equipment expected quantities, unit costs, and replacement frequency for CHWs, supervisors, and managers		
**Medicines**<br>- Expected unit costs and unit mark up for the types of medicine and supplies included in standard treatment guides		
**Capital costs**<br>- Description of assets, expected quantities, replacement frequency, and costs		
**Standard treatment guidelines**<br>- For each service provided by CHWs: description, target population, time per service, and quantities of tests, medicines, and supplies		
**District-level cost efficiencies, geospatial mapping, and analysis (March to June 2019)**		
**Calculated estimates**<br>- Normative cost per CHW service<br>- Number of services per person in target population by type of service in baseline year	Authors	N/A
**Geospatial data**<br>- Geocoded villages; administrative boundaries for health districts, municipalities, and communes; population	N/A	- Mali CHW Mapping tool database<br>- UNOCHA Humanitarian Data Exchange Portal
**Geospatial mapping outputs**<br>- Thiessen polygons for CHW covered villages<br>- Point, choropleth, and Euclidean distance maps	Authors	N/A

Expert Panel Member Profile

1. **Institution represented:** National Directorate of Health

Position: ECC/CHW National Program Manager

Health System Level-Location: Central Government-Bamako

Relevance: Provided design and validation of national ECC/CHW related strategic documents such as *ECC National Strategic Plan 2016-2020* and Implementation Guide. Responsible for overseeing regulatory and normative decisions, overarching coordination and programmatic planning (service expansion, CHW recruitment and deployment) with national and international community health stakeholders.

2. **Institution represented:** National Directorate of Health

Position: ECC/CHW Deputy National Manager

Health System Level-Location: Central Government-Bamako

Relevance: Led data collection field visits with the study team. Program data, including CHW funding source, workforce and service utilization were gathered directly in the regions and from implementing partners.

3. **Institution represented:** Directorate of Pharmacy and Medicine

Position: Quality Assurance Office Chief

Health System Level-Location: Central Government-Bamako

Relevance: Oversees supply chain management, and distribution and quality assurance of medicines for the ECC program. Serves as the liaison authority with regional directorate of health offices for CHW-provided medicines.

4. **Institution represented:** Directorate of Pharmacy and Medicine

Position: Supply Chain and Distribution Officer

Health System Level-Location: Central Government-Bamako

Relevance: Oversees supply chain management, distribution and quality assurance of medicines for the ECC program. Serves as the liaison authority with regional directorate of health offices for CHW-provided medicines.

5, 6, 7. Institution represented: Regional Directorate of Health

Position: ECC/CHW Program Regional Manager

Health System Level-Location: Regional-Sikasso, Regional-Mopti, Regional-Kayes

Relevance: ECC regional managers are dedicated primarily to ECC/CHW activities. They form the technical arm of the MOHSA and are heavily involved in data management and reporting, determination of training needs for CHWs, production and revision of ECC normative documents (e.g. CHW standardized treatment sheet for sick children), training curriculum and supervision norms and guidelines for CHWs, among other things.

8. Institution represented: Regional Directorate of Health

Position: ECC/CHW Regional Manager

Health System Level-Location: Regional-Segou

Relevance: In addition to the general responsibilities of ECC regional managers mentioned above, the study team has received particularly helpful support from the Segou office. A field visit was conducted in Segou in June 2017 with the regional manager of the ECC National Program to directly observe a CHW at work. Although not representative, this visit provided more insights to the study team during expert panel consultative meetings.

9. Institution represented: Regional Directorate of Health

Position: ECC/CHW Program Regional Manager

Health System Level-Location: Regional-Koulikoro

Relevance: In addition to the general responsibilities of ECC regional managers mentioned above, the study team received particularly helpful inputs from the Koulikoro office that had conducted similar work related to estimation of time required. Provision time required for each ECC service was recorded using direct observation of randomly selected CHWs. These inputs contributed to increasing the validity of the estimates coming out of the consultative meetings with the expert panel.

10. Institution represented: District Health Management Team

Position: ECC/CHW Program District Manager

Health System Level-Location: District-Kati

Relevance: Referral health centers are the immediate reference level for CHCs to which CHWs are affiliated. Together with the district management team, they share clinical and public health functions to provide technical supervision of the CHC including CHWs.

11. **Institution represented:** Community Health Center

Position: Technical Director

Health System Level-Location: Communal-Safo Commune

Relevance: Representing the most immediate authority for CHWs and expected to conduct routine supervisions.

Experts were chosen based on their ability to exercise legitimate authority over or influence on one of the following: the choice of CHW location, recruitment and training needs, stakeholder coordination, key program management functions (supply of medicines and equipment, statistical reporting, and supervision), and regulatory and normative decisions. The expert panel information table provides a more detailed understanding of members' profiles looking at the institution and health system level represented, their position and relevance as community health experts. We did not seek individual authorizations from the expert panel members, who will remain anonymous. The table lists only positions and institution represented at the time of the study.

Table S2. ECC Services Costing Outputs

Type of service	Target population	Number of services provided	Number of services needed [1]	Coverage of needs(%) [2]	Unit cost breakdown (US$) Cost per service	CHW labor cost per service	Supply and medicine cost per service [3]	Indirect salary costs[4]	All other indirect costs per service[5]	Total time required to provide services (hours) [6]	Time available spent on service (%) [7]	Total cost in US$ (Share of total cost in %)
**Curative services**												
Curative consultation	Children <5 years	229,593	1,212,487	18.9%	2.38	0.08	0.07	0.50	1.72	57,398	1.31%	545,898 (6.5%)
Malaria rapid screening test	Children <5 years	167,698	533,494	31.4%	3.82	0.10	0.74	0.67	2.29	55,899	1.28%	639,847 (7.7%)
Uncomplicated malaria management	Children <5 years	122,706	3,031,219	4.0%	6.76	0.11	3.38	0.74	2.52	44,992	1.03%	829,141 (9.9%)
Referrals for episode of severe malaria	Children <5 years	10,327	142,666	7.2%	2.31	0.08	0.003	0.50	1.72	2,582	0.06%	23,814 (0.3%)
Diarrhea management	Children 6-59 months	43,584	2,182,476	2.0%	3.39	0.10	0.32	0.67	2.29	14,528	0.33%	147,652 (1.8%)
Pneumonia management	Children 6-59 months	35,601	1,091,238	3.3%	2.75	0.08	0.45	0.50	1.72	8,900	0.20%	97,942 (1.2%)
Cough and cold management	Children 6-59 months	49,728	2,182,476	2.3%	3.90	0.09	1.14	0.61	2.06	14,918	0.34%	194,120 (2.3%)
Moderate acute malnutrition management	Children >1 year to <5 years	36,916	109,858	33.6%	33.84	0.31	24.63	2.02	6.88	36,916	0.85%	1,249,206 (14.9%)
Other curative management	Children <5 years	8,324	1,212,487	0.7%	2.30	0.08	0.00	0.50	1.72	2,081	0.05%	19,172 (0.2%)
**Preventive services**												
Newborn follow-up	Newborns	80,863	1,454,172	5.6%	3.07	0.10	0.003	0.67	2.29	26,954	0.62%	248,554 (3%)
Education on lactational amenorrhea method	Females of reproductive age	12,537	183,622	6.8%	3.84	0.13	0.004	0.84	2.87	5,224	0.12%	48,173 (0.6%)
Oral contraceptives (pill) provision	Females of reproductive age	15,502	227,050	6.8%	4.68	0.13	0.84	0.84	2.87	6,459	0.15%	72,578 (0.9%)

Type of service	Target population	Number of services provided	Number of services needed [1]	Coverage of needs(%) [2]	Unit cost breakdown (US$)					Total time required to provide services (hours) [6]	Time available spent on service (%) [7]	Total cost in US$ (Share of total cost in %)
					Cost per service	CHW labor cost per service	Supply and medicine cost per service [3]	Indirect salary costs[4]	All other indirect costs per service[5]			
Male condom distribution	Females of reproductive age	9,245	138,219	6.7%	1.81	0.05	0.28	0.34	1.15	1,541	0.04%	16,777 (0.2%)
Female condom distribution	Females of reproductive age	188	2,763	6.8%	2.37	0.05	0.84	0.34	1.15	31	0.00%	446 (0.01%)
Standard day method cycle beads distribution	Females of reproductive age	309	752	41.1%	1.86	0.05	0.33	0.34	1.15	52	0.001%	575 (0.01%)
Spermicide-based contraceptives distribution	Females of reproductive age	51	4,526	1.1%	4.33	0.05	2.79	0.34	1.15	9	0.0002%	221 (0.003%)
Provision of contraceptive injections	Females of reproductive age	58,421	855,687	6.8%	1.14	0.03	0.37	0.17	0.57	4,868	0.11%	66,600 (0.8%)
Referrals for family planning	Females of reproductive age	1,332	19,517	6.8%	2.31	0.08	0.003	0.50	1.72	333	0.01%	3,072 (0.04%)
**Promotive services**												
Home visits	Household	120,282	181,203	66.4%	6.15	0.21	0.004	1.35	4.59	80,188	1.84%	739,212 (8.8%)
Health educational talks	Adults >15 years	123,520	1,711,5136	0.7%	9.22	0.31	0.004	2.02	6.88	123,520	2.83%	1,138,438 (13.6%)
Family planning counseling	Females of reproductive age	68,228	501,520	13.6%	2.31	0.08	0.004	0.50	1.72	17,057	0.39%	157,399 (1.9%)
Demand generation	Females of reproductive age	17,127	501,520	3.4%	9.22	0.31	0.004	2.02	6.88	17,127	0.39%	15,7853 (1.9%)
**Monitoring and evaluation**												
Activity reporting	N/A	26,244	N/A	N/A	74.82	2.50	1.12	16.16	55.05	209,952	4.8%	1,963,577 (23.5%)

[1] Expected number of services is based on the prevalence of the different conditions within the target population that would require use of CHW services.
[2] estimated by dividing the actual number of services provided by the expected number of services
[3] An average percentage mark-up on medicines for transport, storage, management, and distribution was determined by the CHW expert panel and applied to each unit cost to account for difficulties in terrain or reach across regions
[4] Indirect salary represents the labor cost for time spent by each CHW outside direct service provision.
[5] Other indirect costs include equipment, labor time for program management and CHW supervision, meetings, training, and supervision visits.
[6]Total time required to provide each service is calculated by multiplying the expected time spent on service (in mn) by the reported service volume for the year. The estimates are then converted in CHW hours.
[7] Percentage of CHW time available spent on services is calculated by dividing the total time required to provide the services reported for the year and the total time available for providing ECC. Availability per year for providing the ECC package is calculated by multiplying 48 hours of CHW standard work time per week by 52 weeks. Time spent on recurrent meetings, training, campaigns, and travel, estimated at 20% of total available work time, was excluded. This information was provided by a national CHW expert panel that included program managers and CHW supervisors from the MOHSA and implementing partners.

Figure S1: Geographic distribution of health facilities in the southern five regions

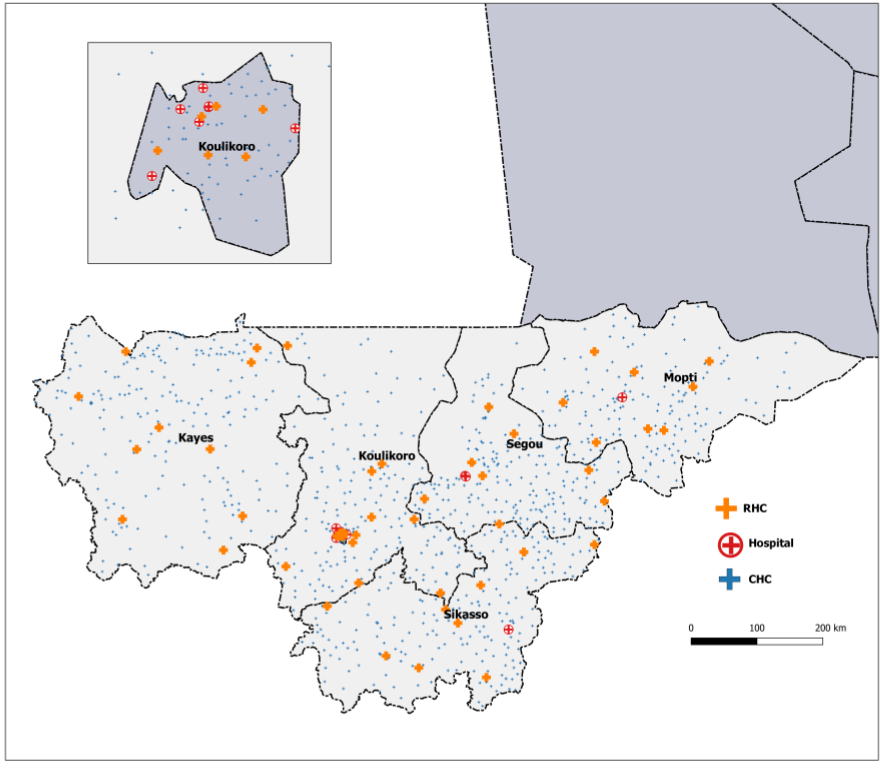

Figure S2: Proximity between CHW-covered villages and distribution of non-covered villages

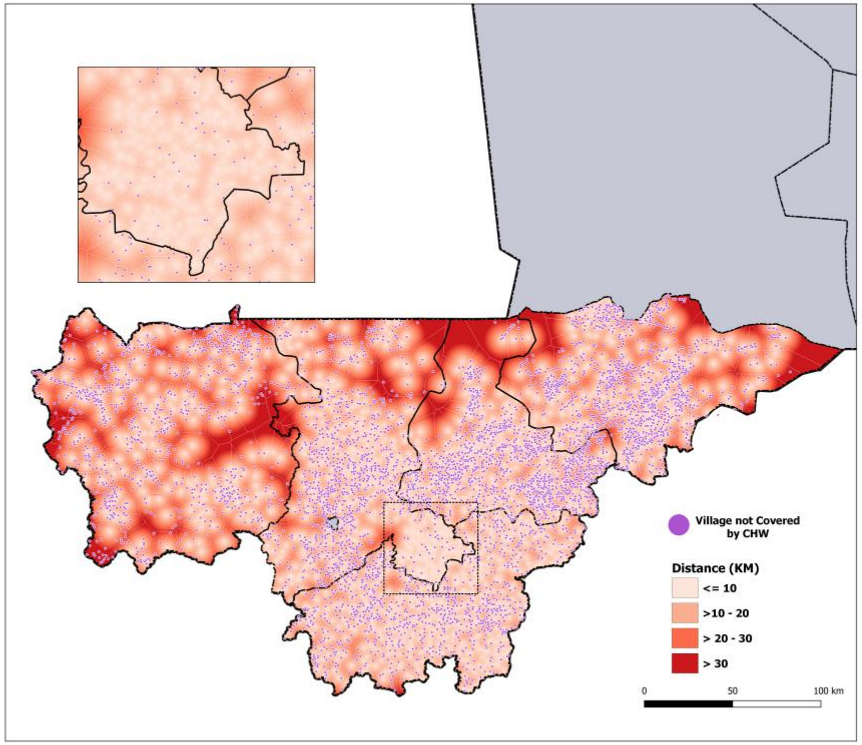

Community health centers are the entry point into the health system, reflected in Figure S1, indicating that there are more CHCs than referral facilities and hospitals. Location data (distance and proximity) used in geospatial targeting can provide useful options on how to expand existing services with funding surplus available for reallocation. Figure S2 provides an additional application of location-based and Euclidean distance mapping analytics. The associated map displays the locations of all non-covered villages and the Euclidean distance—the distance “as the crow flies”—to the nearest covered village. Light areas represent covered villages closer to each other, helping to visually follow CHW program presence across the five regions. These areas can be interpreted as having higher program activity and help visualize the current scale of the program, and how well it reached the population. Some non-covered villages are located in areas benefiting from a higher program presence, and others are over 30 km away from the closest CHW-covered village. Reaching non-covered villages located in areas with higher program presence may be easier to achieve from an operational and logistical standpoint. Such application offers a good example of how stronger program presence/proximity and operations could be leveraged as a way to guide progressive expansion with potentially less distance-based logistical challenges on the supply side. On the demand side, CHW program managers and stakeholders could consider adapting our example to similar Euclidean space analysis used in market segmentation approaches placing groups of consumers/users sharing similar product preferences and characteristics that are located

close to one another. Exploring and extracting these market segments characteristics or preferences from consumer/user data was not, however, the focus of our study and considered beyond its scope.

Table S3: Technical and Allocative Efficiency Gaps

Health District	Rural Population	Covered Population	Number of CHW	Average Spending per CHW	Actual Spending on Covered Population	Normative Cost for Covered Population	Technical Efficiency Surplus (Deficit)	Normative Cost for Rural Population	Allocative Efficiency Surplus (Deficit)
	[A]	[B]	[C]	[D]	[E] = [C] x [D]	[F]	[G] = [E] - [F]	[H] = [F] / [B] x [A]	[I] = [E] - [H]
**Kayes**									
Kayes	210,228	61,361	45	9,213	414,605	196,306	218,299	672,560	(257,955)
Bafoulabe	77,332	33,009	34	9,213	313,257	99,135	214,122	232,249	81,008
Diema	99,199	36,222	27	9,213	248,763	184,383	64,380	504,959	(256,197)
Kenieba	106,732	18,313	14	9,213	128,988	46,319	82,669	269,959	(140,971)
Kita	267,635	82,779	82	9,213	755,502	117,567	637,935	380,108	375,395
Nioro	102,444	14,554	8	9,213	73,708	36,937	36,770	259,999	(186,291)
Yelimane	42,043	14,584	13	9,213	119,775	21,982	97,793	63,370	56,405
Oussoubidiagna	55,676	25,957	25	9,213	230,336	49,890	180,445	107,012	123,324
**Koulikoro**									
Banamba	117,033	97,650	74	4,480	331,542	98,945	232,596	118,585	212,956
Dioila	196,313	109,345	113	4,480	506,273	316,284	189,989	567,841	(61,568)
Fana	127,656	89,678	50	4,480	224,015	157,729	66,286	224,525	(511)
Kalabancoro	113,971	95,146	29	4,480	129,928	70,170	59,758	84,054	45,875
Kangaba	47,764	57,128	36	4,480	161,291	95,109	66,181	79,520	81,771
Kati	222,161	89,895	44	4,480	197,133	28,554	168,578	70,568	126,565
Kolokani	186,546	92,651	49	4,480	219,534	25,312	194,223	50,963	168,571
Koulikoro	119,975	99,642	60	4,480	268,818	44,986	223,831	54,166	214,651

Nara	197,922	47,469	43	4,480	192,653	44,191	148,461	184,256	8,396
Ouelessebougou	147,699	90,678	28	4,480	125,448	84,325	41,123	137,351	(11,903)
**Mopti**									
Bandiagara	204,176	109,965	38	8,329	316,509	122,753	193,756	227,921	88,589
Bankass	184,552	87,899	55	8,329	458,105	225,249	232,856	472,932	(14,826)
Djenne	118,948	77,697	27	8,329	224,888	128,770	96,118	197,137	27,751
Douentza	180,102	60,740	42	8,329	349,826	158,172	191,654	469,000	(119,174)
Koro	162,109	156,524	45	8,329	374,813	134,153	240,661	138,939	235,874
Mopti	150,579	82,683	33	8,329	274,863	97,953	176,910	178,389	96,474
Tenenkou	95,464	47,295	29	8,329	241,546	78,988	162,559	159,435	82,112
Youwarou	85,556	57,458	36	8,329	299,851	100,551	199,300	149,722	150,128
**Segou**									
Segou	301,301	96,546	81	4,414	357,547	173,522	184,025	541,528	(183,981)
Baroueli	108,779	41,085	35	4,414	154,496	89,917	64,579	238,068	(83,573)
Bla	167,030	100,322	47	4,414	207,466	125,238	82,228	208,514	(1,048)
Macina	163,530	100,525	54	4,414	238,365	106,333	132,032	172,978	65,387
Markala	25,757	97,685	74	4,414	326,648	155,812	170,836	41,083	285,565
Niono	154,060	87,426	40	4,414	176,566	102,243	74,323	180,170	(3,604)
San	181,704	100,703	65	4,414	286,921	145,182	141,739	261,960	24,961
Tominian	178,986	88,059	52	4,414	229,536	119,484	110,053	242,859	(13,323)

**Sikasso**										
Bougouni	346,208	151,813	146	4,739	691,943	497,787	194,156	1,135,197	(443,255)
Kadiolo	127,615	53,337	72	4,739	341,232	360,615	(19,383)	862,814	(521,582)
Kignan	91,422	37,969	52	4,739	246,445	170,224	76,221	409,867	(163,422)
Kolondieba	164,598	71,600	81	4,739	383,886	354,668	29,218	815,331	(431,445)
Koutiala	317,519	105,867	92	4,739	436,019	188,376	247,643	564,981	(128,962)
Niena	93,608	35,123	50	4,739	236,967	159,705	77,262	425,636	(188,669)
Selingue	31,132	13,542	18	4,739	85,308	93,259	(7,951)	214,395	(129,087)
Sikasso	245,991	90,253	76	4,739	360,189	250,157	110,033	681,820	(321,631)
Yanfolila	76,355	33,214	35	4,739	165,877	120,490	45,386	276,993	(111,116)
Yorosso	127,896	55,635	38	4,739	180,095	419,313	(239,218)	963,934	(783,839)

Table S3 (rev): Technical and Allocative Efficiency Gaps

Health District	Rural Population	Covered Population	Number of CHW	Average Spending per CHW, US$	Actual Spending on Covered Population, US$	Normative Cost for Covered Population, US$	Technical Efficiency Surplus (Deficit), US$	Normative Cost for Rural Population, US$	Allocative Efficiency Surplus (Deficit), US$
	[A]	[B]	[C]	[D]	[E] = [C] x [D]	[F]	[G] = [E] - [F]	[H] = [F] / [B] x [A]	[I] = [E] - [H]
**Kayes**									
Kayes	210,228	61,361	45	9,213.44	414,604.77	196,305.62	218,299.15	672,559.72	(257,954.96)
Bafoulabe	77,332	33,009	34	9,213.44	313,256.94	99,135.01	214,121.93	232,249.03	81,007.91
Diema	99,199	36,222	27	9,213.44	248,762.86	184,383.31	64,379.55	504,959.41	(256,196.55)
Kenieba	106,732	18,313	14	9,213.44	128,988.15	46,319.44	82,668.71	269,959.42	(140,971.27)
Kita	267,635	82,779	82	9,213.44	755,502.02	117,566.54	637,935.49	380,107.51	375,394.51
Nioro	102,444	14,554	8	9,213.44	73,707.51	36,937.50	36,770.02	259,998.97	(186,291.46)
Yelimane	42,043	14,584	13	9,213.44	119,774.71	21,981.85	97,792.86	63,369.65	56,405.06
Oussoubidiagna	55,676	25,957	25	9,213.44	230,335.98	49,890.50	180,445.48	107,011.73	123,324.26
**Koulikoro**									
Banamba	117,033	97,650	74	4,480.29	331,541.59	98,945.10	232,596.49	118,585.17	212,956.42
Dioila	196,313	109,345	113	4,480.29	506,272.97	316,283.59	189,989.37	567,841.06	(61,568.09)
Fana	127,656	89,678	50	4,480.29	224,014.59	157,728.56	66,286.02	224,525.49	(510.91)
Kalabancoro	113,971	95,146	29	4,480.29	129,928.46	70,170.42	59,758.04	84,053.90	45,874.56
Kangaba	47,764	57,128	36	4,480.29	161,290.50	95,109.41	66,181.09	79,519.78	81,770.73
Kati	222,161	89,895	44	4,480.29	197,132.84	28,554.41	168,578.43	70,567.62	126,565.22
Kolokani	186,546	92,651	49	4,480.29	219,534.30	25,311.73	194,222.57	50,963.31	168,570.99
Koulikoro	119,975	99,642	60	4,480.29	268,817.50	44,986.19	223,831.32	54,166.09	214,651.41
Nara	197,922	47,469	43	4,480.29	192,652.54	44,191.41	148,461.13	184,256.09	8,396.45
Ouelessebougou	147,699	90,678	28	4,480.29	125,448.17	84,325.23	41,122.94	137,351.41	(11,903.25)
**Mopti**									
Bandiagara	204,176	109,965	38	8,329.19	316,509.04	122,753.30	193,755.74	227,920.51	88,588.54
Bankass	184,552	87,899	55	8,329.19	458,105.20	225,249.33	232,855.87	472,931.59	(14,826.39)

Djenne	118,948	77,697	27	8,329.19	224,888.00	128,770.33	96,117.67	197,137.26	27,750.75
Douentza	180,102	60,740	42	8,329.19	349,825.79	158,171.67	191,654.11	468,999.58	(119,173.80)
Koro	162,109	156,524	45	8,329.19	374,813.34	134,152.64	240,660.71	138,939.39	235,873.95
Mopti	150,579	82,683	33	8,329.19	274,863.12	97,953.27	176,909.84	178,388.62	96,474.50
Tenenkou	95,464	47,295	29	8,329.19	241,546.38	78,987.59	162,558.79	159,434.85	82,111.52
Youwarou	85,556	57,458	36	8,329.19	299,850.67	100,551.13	199,299.54	149,722.45	150,128.22
**Segou**									
Segou	301,301	96,546	81	4,414.16	357,547.15	173,522.07	184,025.09	541,528.10	(183,980.95)
Baroueli	108,779	41,085	35	4,414.16	154,495.68	89,916.55	64,579.14	238,068.20	(83,572.51)
Bla	167,030	100,322	47	4,414.16	207,465.63	125,238.08	82,227.55	208,513.76	(1,048.13)
Macina	163,530	100,525	54	4,414.16	238,364.77	106,332.76	132,032.01	172,977.83	65,386.94
Markala	25,757	97,685	74	4,414.16	326,648.02	155,811.55	170,836.46	41,083.46	285,564.55
Niono	154,060	87,426	40	4,414.16	176,566.50	102,243.14	74,323.36	180,170.41	(3,603.91)
San	181,704	100,703	65	4,414.16	286,920.55	145,181.84	141,738.71	261,959.64	24,960.92
Tominian	178,986	88,059	52	4,414.16	229,536.44	119,483.86	110,052.59	242,859.19	(13,322.75)
**Sikasso**									
Bougouni	346,208	151,813	146	4,739.33	691,942.77	497,786.61	194,156.16	1,135,197.29	(443,254.52)
Kadiolo	127,615	53,337	72	4,739.33	341,232.05	360,615.42	(19,383.37)	862,814.49	(521,582.44)
Kignan	91,422	37,969	52	4,739.33	246,445.37	170,224.26	76,221.11	409,867.05	(163,421.68)
Kolondieba	164,598	71,600	81	4,739.33	383,886.06	354,668.33	29,217.73	815,330.97	(431,444.91)
Koutiala	317,519	105,867	92	4,739.33	436,018.73	188,375.63	247,643.10	564,980.97	(128,962.24)
Niena	93,608	35,123	50	4,739.33	236,966.70	159,704.50	77,262.20	425,636.16	(188,669.46)
Selingue	31,132	13,542	18	4,739.33	85,308.01	93,259.01	(7,951.00)	214,395.17	(129,087.16)
Sikasso	245,991	90,253	76	4,739.33	360,189.39	250,156.81	110,032.58	681,820.26	(321,630.87)
Yanfolila	76,355	33,214	35	4,739.33	165,876.69	120,490.36	45,386.33	276,992.87	(111,116.18)
Yorosso	127,896	55,635	38	4,739.33	180,094.69	419,312.97	(239,218.27)	963,933.70	(783,839.01)
